# Retraction Note: TFPI-2 suppresses breast cancer cell proliferation and invasion through regulation of ERK signaling and interaction with actinin-4 and myosin-9

**DOI:** 10.1038/s41598-025-20055-0

**Published:** 2025-09-17

**Authors:** Guangli Wang, Wenhe Huang, Wei Li, Shaoying Chen, Weibin Chen, Yanchun Zhou, Pei Peng, Wei Gu

**Affiliations:** 1 Department of Pathophysiology, The Key Immunopathology Laboratory of Guangdong Province, Shantou University Medical College, Shantou, Guangdong Province 515041 China; 2 Tumor Hospital, Shantou University Medical College, Shantou, Guangdong Province 515041 China; 3 Xiang’an Hospital of Xiamen University, Xiamen, Fujian Province 361101 China; 4 Present Address: Department of Prepotency and Genetics, Affiliated Hospital of Guilin Medical University, Guilin, Guangxi 541001 China

Retraction of: *Scientific Reports* 10.1038/s41598-018-32698-3, published online 26 September 2018

The Editors have retracted this Article.

After publication, concerns were raised regarding the Western blot data presented in this Article. Specifically:

- a portion of the *MYH9* gel slice in Figure 5D appears to overlap, when horizontally flipped and re-scaled, with a portion of the *Beta-actin* band in Figure 7A;
- the *pEGFR* gel slice in Figure 2A and the *TFPI-2* gel slice in Figure 3D appear to have been edited.

The Authors did not reply to repeated requests from the Editors for the source data. The Editors therefore no longer have confidence in the results and conclusions reported.

The Authors did not reply to correspondence from the Editors about this retraction.

